# A rare mediastinal occurrence of neuroblastoma in an adult: case report

**DOI:** 10.1590/1516-3180.2017.0160140617

**Published:** 2018-03-05

**Authors:** Fazli Yanik, Yekta Altemur Karamustafaoglu, Yener Yoruk

**Affiliations:** I MD. Assistant Professor, Department of Thoracic Surgery, Trakya Üniversitesi Tip Fakültesi, Edirne, Turkey.; II MD. Associate Professor, Department of Thoracic Surgery, Trakya Üniversitesi Tip Fakültesi, Edirne, Turkey.; III MD. Professor, Department of Thoracic Surgery, Trakya Üniversitesi Tip Fakültesi, Edirne, Turkey.

**Keywords:** Mediastinum, Neuroblastoma, Adult

## Abstract

**CONTEXT:**

Neuroblastoma is the most common extracranial malignant solid tumor that occurs during childhood. It arises from primitive cells and is seen in the adrenal medulla and sympathetic ganglia of the sympathetic nervous system.

**CASE REPORT:**

We present a rare case of a 40-year-old man who was diagnosed with the onset of neuroblastoma arising in the mediastinum. He was treated by means of surgical resection in the superior mediastinum after neoadjuvant chemotherapy. The patient’s surgical outcome was satisfactory.

**CONCLUSION:**

There are still no standard treatment guidelines for adult neuroblastoma patients. Although they have a poor prognosis, the main treatment option should be complete surgery at an early stage. This situation may become clarified through biological and genetic studies in the future.

## INTRODUCTION

Neuroblastomas are the most common extracranial malignant solid tumors that occurs during childhood. They arise from primitive cells and are seen in the adrenal medulla and sympathetic ganglia of the sympathetic nervous system. These aggressive cells begin to grow uncontrollably. Neuroblastomas may lead to signs such as swelling in the face, neck, arms and upper chest, headaches, dizziness, changes to consciousness, drooping eyelids and small pupils. They may also lead to signs of paraneoplastic syndromes that include constant diarrhea, fever, high blood pressure (causing irritability), rapid heartbeat, flushing of the skin and sweating. Because neuroblastomas consist of embryonic cells, they are especially common among small children: up to 90% of the patients are younger than six years old. On the other hand, neuroblastomas are only rarely seen in older children and adults. Incidence rates of one case per 100,000 children per year and one case per 10 million adults per year have been reported.^1^^,^^2^ The most common locations in adults are the chest, pelvis and neck.^2^ Approximately 20% of the cases occur in the mediastinum.^2^

Multimodal treatments are used, including surgery, chemotherapy and radiotherapy. No standard treatment protocol has been developed for adults. Thus, the same protocol is used for adults and children. In adults, the disease presents much more aggressively than in children.^2^ Here, we present a rare case of neuroblastoma in an adult, which was treated by means of neoadjuvant chemotherapy and surgery.

## CASE REPORT

**Figure 1:**
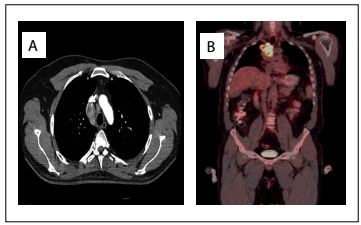
A) Computed tomography (CT) scan showing that the anterior mediastinal tumor invaded the major mediastinal structures. B) Positron-emission tomography (PET)-CT scan showing an aggressive mediastinal mass with high 18-FDG (fluorodeoxyglucose) affinity.

A 40-year-old male patient was hospitalized with complaints of anorexia, fatigue, headache and weight loss. Physical examination and routine blood tests were unremarkable. Chest x-ray detected a right paratracheal lesion. The following tumor markers were all negative: carcinoembryonic antigen, carbohydrate antigen 19-9, alpha fetoprotein and vanillylmandelic acid, as also were urine tests. Lactate dehydrogenase and neuron-specific enolase blood levels were also within normal limits. Catecholamine metabolites were not found to be elevated in 24-hour urine collection. Chest computed tomography (CT) examination revealed a mass of lobulated outline between the lower border of the superior vena cava and the subcarinal area (Figure 1A and 1B). Adjacent vascular and mediastinal structures had been invaded. Its size was 5 cm x 4 cm. In positron-emission tomography (PET)-CT images, the standardized uptake value was 19.5.

Therefore, he underwent mediastinoscopy for diagnostic purposes. The histopathological findings revealed the presence of a neuroblastoma. The morphology comprised small round cells. The absence of ganglion cells and state of maturation determined that the neuroblastoma was of poorly differentiated stroma-poor Schwannian subtype. The Ki-67 proliferation rate was 70%. The tumor cells showed a diffuse strong reaction with neuron-specific enolase. A bone marrow biopsy confirmed that this had not become infiltrated by tumor cells.

The patient was diagnosed as having stage III unresectable neuroblastoma. The multidisciplinary oncology council decided to implement three cycles of neoadjuvant chemotherapy. These three cycles, which were repeated every 14 days, consisted of a regimen of ifosfamide (day 1; 5000 mg/m^2^), carboplatin [day 1; optimized to achieve the area under the curve (AUC) dose calculation = 5; maximum of 800 mg] and etoposide (days 1-3; 100 mg m^2^) (ICE regimen).

**Figure 2:**
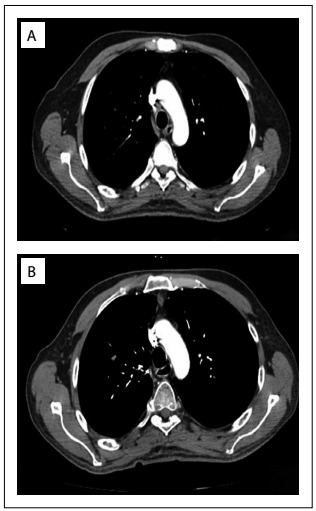
A) Computed tomography (CT) scan after neoadjuvant chemotherapy, showing that the tumor had almost completely regressed. B) CT scan showing absence of recurrence three months after surgery.

Contrast-enhanced chest CT was performed after chemotherapy and revealed that the tumor had almost completely regressed. Therefore, an operation was planned (Figure 2A). Muscle-sparing right posterolateral thoracotomy was performed. The tumor was found to have become attached to the mediastinal structures, especially the superior vena cava and trachea. Nonetheless, it was possible to completely resect the tumor by means of blunt and sharp dissection without causing any complications. Lymph node dissection was added to the surgery.

Histopathological examination showed the presence of necrotic tumor tissue. Metastasis was only detected in hilar lymph nodes. No complications were encountered during the postoperative period. A chest CT scan performed three months later did not show any recurrence (Figure 2B).

## DISCUSSION

Neuroblastoma is a very common childhood disease, but it is rarely detected in adolescents and adults. The most important clinically relevant factors that influence survival among these patients are stage, age, histology and tumor grade. Young age and low stage with timely diagnosis are two important favorable prognostic features.^3^ Whereas five-year overall survival is 85% for infants, it is only 36% for adults.^2^

Currently, there are no standard treatment guidelines for patients with adult neuroblastoma. Thus, attempts to adapt treatment protocols developed for children have been made, for use in adult cases.^4^ Different treatments are required according to different stages of the disease. Localized tumors are treated by means of primary surgery if possible (stages 1 and 2). Neoadjuvant chemotherapy (NCT) is recommended in cases of inoperable stage 3 neuroblastoma. Local radiotherapy may be indicated for aggressive tumors, with or without total resection of the primary tumor. Metastatic neuroblastoma (stage 4) requires neoadjuvant chemotherapy followed by surgery of the primary tumor, if possible. Stage 4S may regress spontaneously. However, half of these cases need chemotherapy and radiotherapy because of tumor progression. Some localized or stage 4S tumors may even show spontaneous regression without any treatment.^3^^,^^4^ Our case was diagnosed as having stage 3 neuroblastoma. It was unresectable. However, it became possible to completely resect the tumor after it regressed, through NCT.

Tumors categorized as neuroblastoma have been further divided into three subtypes: undifferentiated, poorly differentiated and differentiated, based on their degree of neuroblastic differentiation.^4^ Presence of Schwannian stroma in neuroblastomas is related to patient prognosis. Our case was identified as the poorly-differentiated stroma-poor Schwannian subtype.

Conter et al.^5^ compared neuroblastoma cases in 118 adults (mean age of 47 years) and 112 children (mean age of 5 years). For all stage-matched categories, the prognoses for the adult patients were not statistically different from those of the pediatric neuroblastoma patients.

A search of the literature in major medical databases for case reports on neuroblastoma, neuroblastoma in adults and mediastinal neuroblastoma is presented in Table 1.

**Table 1:** Search of the literature in medical databases for case reports on neuroblastoma, neuroblastoma in adults and mediastinal neuroblastoma treated by means of thoracic surgery. The search was conducted on May 22, 2017

Database	Search strategies	Papers found	Related papers
MEDLINE (via PubMed)	((((Neuroblastoma AND Neuroblastoma in adult) AND Mediastinal neuroblastoma) AND “case reports” [Publication Type]	6	2
Embase (via Elsevier)	((((Neuroblastoma AND Neuroblastoma in adult) AND Mediastinal neuroblastoma) AND “case reports” [Publication Type]	8	0
LILACS (via Bireme)	((Neuroblastoma AND Neuroblastoma in adult) AND Mediastinal neuroblastoma) AND “case reports” [Publication Type]	4	1

## CONCLUSION

The diagnosis of neuroblastoma in adults has been reported in few case reports. There are still no standard treatment guidelines for adult neuroblastoma patients. The main treatment option should be complete surgery at an early stage. At advanced stages, multimodal oncological treatment can be performed, followed by surgery, if this is possible. It is unclear why these patients have a poor prognosis. This situation may become clarified through biological and genetic studies in the future.
